# Puerperal Fever

**Published:** 1874-10

**Authors:** 


### PUERPERAL FEVER.

After discussing in a clinical lecture some of the views with regard to the nature of puerperal fever, Professor Barker states that his "own belief is that it is a special poison characterized by certain general phenomena which always belong to the disease, with anatomical lesions which take the epidemical type of the seasons or of the locality in which it occurs; that it may be, or it may not be, accompanied by any of the local lesions; that it differs from inflammations in its nature and its results, but any and all of the inflammations may be present."

"We are aware that it produces purulent deposits, and if they have an external manifestation anywhere, it is to be regarded as an effort of the system to eliminate the poisonous element, and the prognosis is more favorable; but when the deposits take place in the internal organs, it must be inevitably fatal; that it may be, or or may not be, accompanied by septicemia; but more frequently, in prolonged cases such as those which last for five or eight days, it is accomponied with septicemia, but in other cases it is not."

The principal reasons for these views, which are contrary to the current opinions of the day, may be embraced under four heads:

1st. That puerperal fever has no special characteristic anatomical lesions.

2d. The lesions which exist are not sufficient to explain the cause of death or the progress of the disease.

3d. We may have the most intense inflammatiou of the pelvic organs, and the disease not be puerperal fever.

4th. The lesions differ essentially from those produced by inflammation.

"The characteristic features of puerperal fever, wherever it occurs, are a morbid increase in temperature, increase in the frequency of pulse, as well as change in character, and increased frequency of respiration. Any puerperal woman who shows a temperature over 100°, a pulse over 100 to the minute, and a respiration over 24 to the minute must be considered as being in an abnormal condition; and these phenomena ofttimes appear before any othei symptoms which will attract attention. Puerperal fever is not invariably ushered in by a chill, for some of the most fatal cases are free from chills during the entire course of the disease. Pain and abdominal tenderness are unreliable symptoms in this disease. Tympanitis, also, is not characteristic. It is one of the most treacherous diseases you will ever be called upon to treat. The patient herself may say that she is better; there may be less pain, if pain has been prominently present; there may be less tympanitis, fever may have subsided, and desire for food returned, and the patient may even have a good appetite just before death."

With regard to treatment, Prof. Barker recommends such doses of opium as may be required to hold the pain in complete subjection, and it is sometimes astonishing to what extent this drug may be used without transcending the point of tolerance. The only rule which can be given with regard to its administration is to completely control all pain and nervous irritation. It is well to commence by giving such quantities as are represented by five or ten-drop doses of Majendie's solution, once an hour or two hours, and continue, it may be, to a point just short of producing narcotism. In cases where there is apparently no peritoneal lesion, but where the secondary lesion is chiefly phlebitis or metro-phlebitis, quinine proves very valuable in doses of ten to fifteen grains, morning and evening. We are able to accomplish but very little in the way of elimination. To control the vascular and nervous system, the tincture of veratrum viride is superior to all other agents. It will reduce the pulse when the excitement is due to an inflammatory process, with an almost absolute certainty. It may be given in doses of five drops, once every hour or two, increasing to eight or ten, until we bring the pulse from 120 or 130 to 80, if possible. In a very large majority of cases it can be brought down to this point, but in some we cannot get it below 100. Quinine is of great value to control the tendency to suppuration, in doses of ten or fifteen grains, morning and evening, or even larger. The tendency, if any such exist, to produce paralysis of the motor power of the heart may be obviated by giving half-ounce doses of whisky, once in three or four hours. By combining fifteen or twenty grains of the bromide of potassium with each dose of the quinine, much larger doses can be given without unpleasant symptoms.—*The Medical Record,* July.

